# RAGE Mediates Accelerated Diabetic Vein Graft Atherosclerosis Induced by Combined Mechanical Stress and AGEs via Synergistic ERK Activation

**DOI:** 10.1371/journal.pone.0035016

**Published:** 2012-04-09

**Authors:** Yuhuang Li, Shuying Liu, Zhengyu Zhang, Qingbo Xu, Fukang Xie, Jingjing Wang, Suning Ping, Chen Li, Zhaojing Wang, Min Zhang, Jintao Huang, Dadi Chen, Liping Hu, Chaohong Li

**Affiliations:** 1 Department of Histology and Embryology, Zhongshan School of Medicine, Sun Yat-sen University, Guangzhou, China; 2 Cardiovascular Division, King's College London British Heart Foundation Centre, London, United Kingdom; University of Pittsburgh, United States of America

## Abstract

**Aims/Hypothesis:**

Diabetes with hypertension rapidly accelerates vascular disease, but the underlying mechanism remains unclear. We evaluated the hypothesis that the receptor of advanced glycation end products (RAGE) might mediate combined signals initiated by diabetes-related AGEs and hypertension-induced mechanical stress as a common molecular sensor.

**Methods:**

*In vivo* surgical vein grafts created by grafting vena cava segments from C57BL/6J mice into the common carotid arteries of streptozotocin (STZ)-treated and untreated isogenic mice for 4 and 8 weeks were analyzed using morphometric and immunohistochemical techniques. *In vitro* quiescent mouse vascular smooth muscle cells (VSMCs) with either knockdown or overexpression of RAGE were subjected to cyclic stretching with or without AGEs. Extracellular signal-regulated kinase (ERK) phosphorylation and Ki-67 expression were investigated.

**Results:**

Significant increases in neointimal formation, AGE deposition, Ki-67 expression, and RAGE were observed in the vein grafts of STZ-induced diabetic mice. The highest levels of ERK phosphorylation and Ki-67 expression in VSMCs were induced by simultaneous stretch stress and AGE exposure. The synergistic activation of ERKs and Ki-67 in VSMCs was significantly inhibited by siRNA-RAGE treatment and enhanced by over-expression of RAGE.

**Conclusion:**

RAGE may mediate synergistically increased ERK activation and VSMC proliferation induced by mechanical stretching with and without AGEs. It may serve as a common molecular bridge between the two, accelerating vascular remodeling. This study provides potential drug targets and novel therapeutic strategies for the treatment of vascular diseases resulting from diabetes with hypertension.

## Introduction

Diabetes and hypertension are independent risk factors of atherosclerosis. However, up to 70% of patients with type 2 and up to 40% of patients with type 1 diabetes have arterial hypertension [1], [2]. The combination of diabetes and hypertension may amplify or accelerate the development and progression of atherosclerosis [3], [4], [5]. There may be a common cut-in point or pathway between diabetes and hypertension related to the accelerated vascular remodeling. If so, it would be very valuable in the treatment of diabetes with and without hypertension.

Diabetes-related vascular injury is closely associated with the deposition of advanced glycation end products (AGEs) as a result of prolonged hyperglycemia through non-enzymatic reactions between glucose and long-lived proteins (e.g. vessel wall collagen), lipids, and nucleic acids in plasma and tissues [6], [7], [8]. These modified proteins interact with AGE receptor (RAGE) to initiate intracellular signaling, e.g. extracellular signal-regulated kinase (ERK) activation [9]. This triggers altered vascular structure and function, which accelerates the progression of atherosclerosis and hypertension in diabetic patients or animals [10], [11], [12], [13], [14], [15], [16].

Once hypertension occurs, hypertension-induced abnormal biomechanical stretching becomes the predominant stimulus [17], [18], [19]. Hypertension increases cyclic strain stress on the arterial walls by as much as 30%, which induces VSMC hypertrophy and hyperplasia [17], [18], [19], [20], [21], [22], [23]. This leads to continuously elevated peripheral vascular resistance and the formation of macrovascular neointima. The saphenous vein conduits used for coronary artery bypass surgery (CABG) in patients with ischemic heart disease also endure rapidly increased arterial pressure resulting in vein graft occlusive disease in nearly half of the conduits within 10 years. Patients with diabetes are particularly at risk [20]. This implies that arteries and vein bypass grafts in diabetic subjects with hypertension experience combined stimulation from AGEs due to prolonged hyperglycemia and cyclic stretching induced by increased blood pressure [6], [7], [15], [16], [17], [18], [19], [21], [22], [23], [24], [25]. To date, the underlying mechanism by which this combined stimulation triggers accelerated vascular remodeling remains unclear [5], [26].

Murine venous bypass graft atherosclerosis *in vivo* and cell signals induced by mechanical cyclic stretching *in vitro* are two very important models, which permit mechanistic study of neointimal formation and its relationship with hypertension with and without different metabolic disorders [15], [22]. Certain well-known chemical materials (e.g. hormones, AGE, and drugs) can specifically bind to their receptors, triggering activation of individual signal pathways. However, to date, no data are available regarding the presence of any specific mechanoceptors existing in cells in response to mechanical stress. The means by which the vascular cells sense and transduce mechanical stimulation into intracellular biochemical signals and which receptors mediate those signals remains unclear.

Several previous studies have indicated that some transmembrane receptors e.g. PDGFα-receptor in VSMCs, angiotensin II type 1 receptor in cardiocytes, and FLK-1 and integrins in endothelial cells can be directly activated by mechanical stress as mechanoceptors [23], [27], [28], resulting in increased ERK phosphorylation and cell proliferation [29]. Assuming that RAGE is one kind of transmembrane receptor present in VSMCs, we proposed that RAGE would be a common sensor, capable of simultaneously mediating signals induced by mechanical stretching and AGEs, contributing to vascular remodeling in patients with diabetes and hypertension. In the present study, we determined the impact of diabetes with hypertension on vein graft neointimal formation and that its mechanism involves the RAGE/ERK signal pathway. This study provides new targets for drug development and new strategies for the prevention and treatment of vascular diseases in diabetes patients with hypertension.

## Methods

An expanded methods section is available in Data S1.

### Experimental animals

All animal procedures were consistent with the National Institutes of Health Guide for the Care and Use of Laboratory Animals and approved by the Animal Care and Use Committee of Sun Yat-sen University and similar to the protocols described previously [25], [29], [30], [31]. In brief, three-month-old male C57BL/6J mice were purchased from the animal facility center of Sun Yat-sen University (Guangzhou, China) and maintained on a light/dark (12/12 h) cycle at 24°C and received food and water ad libitum before experimentation.

### Vein grafting of diabetic mice in vivo

The mice were used as donors and recipients for vein grafts (N = 50, respectively) and divided into a non-diabetic vein graft group and a diabetic vein graft group. The induction of experimental diabetes was similar to that described by Zauli [30]. The recipients received seven consecutive daily intraperitoneal injections of 50 mg/kg streptozotocin (STZ) (Sigma) (diabetic mice, D mice) or citrate buffer (nondiabetic mice, ND mice) (N = 25, respectively). Blood glucose concentrations were measured a week later and hyperglycemia (blood glucose level >16 mmol/L) was confirmed in the diabetic group (N = 24). The mice were subjected to vein graft surgery in a manner similar to that described previously [25], [32]. In brief, the right common carotid artery of the recipient was mobilized from the thoracic inlet to the bifurcation, divided at its midpoint, inverted over the polyethylene cuff and fixed with 8-0 silk sutures. The supradiaphragmatic vena cava from an isogenic littermate donor mouse was harvested and sleeved over the 2 cuffs and ligated as an interposition graft. Vigorous pulsations confirmed successful engraftment.

For histological analysis, perfusion was performed as described previously [25]. After the mice were placed under anesthesia using pentobarbital sodium (50 mg/kg body weight, i.p.), blood was collected from each mouse's left atrium for AGE measurement by spectroscopy [33] (Varian, California, U.S.). Samples were perfusion-fixed with 0.9% NaCl and 4% paraformaldehyde. The vein grafts were harvested at 0, 4, and 8 weeks after the operation and paraffin-embedded (N = 7, respectively) [25]. Sample sections 7 mm thick were stained with hematoxylin and eosin (HE) and examined microscopically (Carl Zeiss, Oberkochen, Germany). The thickness of the grafted vessel wall, including the distance between the lumen intima and adventitia, was determined by measuring four regions of each cross-section.

### Immunohistochemical staining

Procedures were in accordance with the protocols provided by Abcam (www.abcam.com/technical). Briefly, serial paraffin-embedded sections were stained with α smooth muscle actin antibody (1∶200, Sigma-Aldrich, St. Louis, U.S.), AGE antibody (1∶200, Abbiotec, San Diego, U.S.), or RAGE antibody (1∶100, Santa Cruz, California, USA) or Ki-67 antibody (1∶100, Santa Cruz) or phosphorylated ERK1/2 (pERK1/2) antibody (1∶200, Cell Signal Tech., Inc., U.S.). The sections incubated with horseradish peroxidase (HRP)-conjugated secondary antibody were developed with chromogen (3,3′-diaminobenzidine, DAB) (brown) and counterstained with hematoxylin (blue). They were then inspected and photographed using visible light microscopy (Carl Zeiss, Oberkochen, Germany). The sections with TRITC-conjugated secondary antibody were counterstained with 4′, 6-diamidino-2-phenylindole (DAPI) (blue). They were inspected and photographed using fluorescence microscopy (Olympus, Tokyo, Japan). Nuclei and Ki-67-positive cells were counted and analyzed. Active proliferating cells were identified by Ki-67-positive staining, and the proliferation index was calculated as the percentage of active proliferating cells versus the total cell count.

### Cell culture

VSMCs were isolated by enzymatic digestion of aorta of C57BL/6J mice using a modification of the procedure as described previously [29], [31]. The isolated cells grown in silicone elastomer-bottomed, gelatin-coated 6-well culture plates were cultured in Dulbecco's modified Eagle's medium (DMEM) (Life Technologies, California, U.S.) supplemented with 10% fetal calf serum, penicillin and streptomycin at 37°C in a humidified atmosphere of 5% CO_2_.

### AGE preparation

AGEs were prepared in a manner similar to that described by Kim [34]. In brief, 1 mM fatty acid-free BSA was dissolved in PBS with 0.5 M glucose and incubated under sterile condition for 8 weeks at 37°C. Reaction mixtures were dialyzed against phosphate-buffered saline (PBS) to remove free glucose and then passed through a specific column (Pierce, Illinois, U.S.) to remove any endotoxins. Non-glycated control BSA was subjected to the same conditions except that glucose was omitted. AGEs were identified by fluorescence spectrophotometry [33].

### Cyclic strain stress

Treated VSMCs were subjected to cyclic stretch stress as described previously [29], [35]. Serum-starved VSMCs achieving 70% confluence were subjected to cyclic stretch stress with a computer-controlled cyclic stress unit [35]. Cyclic deformation (60 cycles/min) achieving 5% to 20% elongation in elastomer-bottomed plates was performed with and without AGEs.

### Cell treatment

RAGE small interfering RNA (siRNA-RAGE)-treated VSMCs were serum-starved and subjected to cyclic stretching with and without AGEs. They were then harvested for Western blot analysis and immunocytochemical staining to assess of Ki-67 expression (see below). We used procedures provided by Origene to establish stable VSMC lines expressing RAGE. Resistant monoclonal cells selected by G418 were used for the corresponding experiment.

### Western blot analysis

Procedures were similar to those described previously, with slight modifications [29]. Treated VSMCs were harvested in lysis buffer using protease inhibitors. The lysate suspension was centrifuged and protein concentration was assessed using a Bio-Rad protein assay. Heat-denatured proteins were resolved by SDS-PAGE and electrophoretically transferred onto nitrocellulose membranes. These were probed with antibodies against phosphorylated ERK1/2 (pERKs) and RAGE and reprobed with β-actin antibody. The bands were visualized using the enhanced chemiluminescence (ECL) detection system. The intensity was quantitated using densitometry.

### RNA interference

The procedures used for this experiment were similar to that described by Villacorta [36]. The RAGE small interfering RNA (siRNA-RAGE) target duplex sequences [NM_007425] (Sense: 5′- GAGACACCCUGAGACGGGACUCUUU-3′; Antisense: 5′- AAAGAGUCCCGUCUCAGGGUGUCUC-3′) were synthesized by Invitrogen (Carlsbad, U.S.). A non-targeting siRNA duplex sequence (Invitrogen Stealth™ RNAi) was used as a negative control. VSMCs transfection was performed according to the manufacturer's recommendations. Serum-starved VSMCs were subjected to cyclic stretch stress in the absence or presence of AGEs for 10 minutes for Western blot analysis or for 1 hour. They were cultured for an additional 24 hours for immunocytochemical staining.

### Statistical analysis

All analyses were performed with SPSS 16.0 (SPSS Inc, Chicago, U.S.). Continuous variables are given as mean ± SEM and categorical variables are given as actual numbers and percentages. ANOVA was used for continuous variables and chi-square and Fisher exact tests were used for categorical variables. There were no missing values, and P values were adjusted for multiple comparisons of data with either the Scheffé or modified Bonferroni method. P values<0.05 were considered significant.

## Results

### Diabetic vein grafted model

Blood glucose levels in STZ-injected mice (D mice) were significantly increased compared to those in citrate buffer-injected mice (ND mice) (blood glucose in D mice with vein grafts at 0, 4, and 8 weeks were 23.53±1.29, 25.56±1.84, and 26.26±1.34 versus time-matched ND mice 6.80±0.76, 6.91±0.92, and 6.74±0.79, respectively, P<0.05). Blood glucose levels showed a slight increase over time in D mice but there were no significant differences. Twenty-four mice from the D group and the same number of mice from the ND group received a vein-grafted operation. Turgor vitalis and vigorous pulsations of vein grafts confirmed successful engraftment. At the point of euthanasia, data from surviving mice in each group (N = 7) were collected for statistical analysis.

### Effects of combined increases in blood pressure and blood glucose on neointima formation in mouse vein grafts

**Figure 1:**
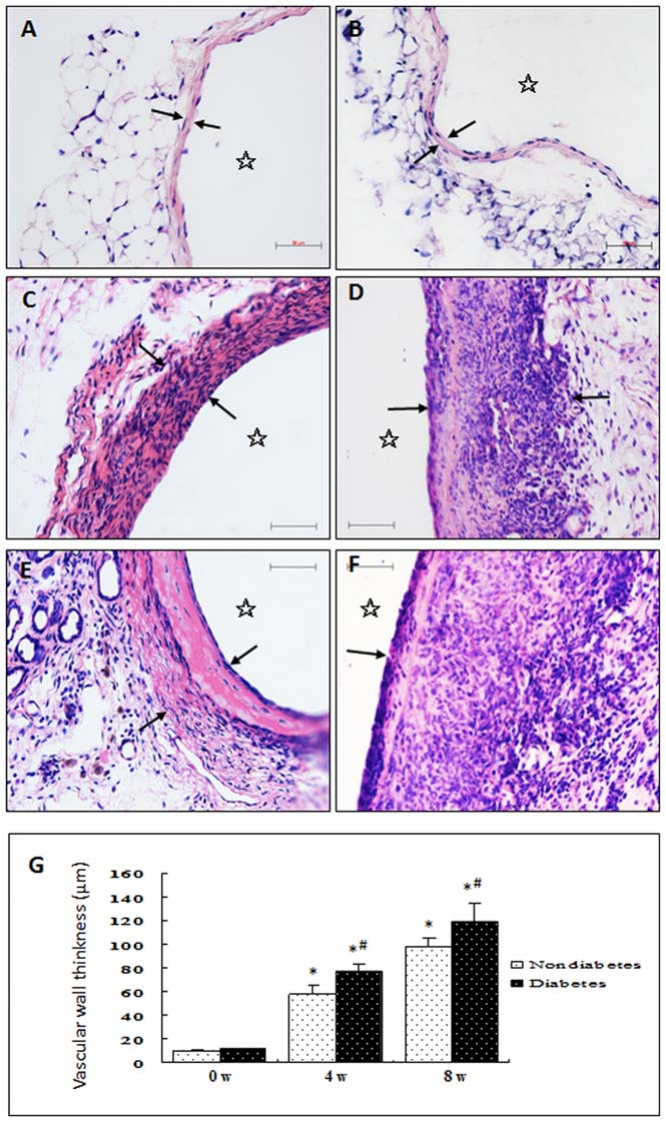
Representative photomicrograph of HE-stained sections of mouse control veins and vein grafts. Under anesthesia, the vena cava segments of mice were grafted into the carotid arteries of (A, C, E) nondiabetic and (B, D, F) diabetic mice. Animals were killed (A, B) 0, (C, D) 4, and (E, F) 8 weeks after surgery, and the grafted veins were fixed in 4% phosphate-buffered (pH 7.2) formaldehyde, embedded in paraffin, sectioned, and stained with HE. Arrowheads and stars indicate the wall thickness and lumens, respectively, of the (A, B) control vessel and (C–F) vein grafts. G shows statistical graphs of wall thickness of vein grafts of different groups (0, 4, and 8 weeks after operation). *P<0.05 *versus* normal control of ND mice, #P<0.05 *versus* time-matched ND groups. Bar = 50 µm.

Histological examination of all vein graft cross-sections was performed on HE-stained sections. Each subject showed a complete and circumferentially viable vein graft with a patent lumen. The vena cava of both ND and D mice showed that normal vein structure consists of intima (a monolayer of endothelial cells), media (a single or double layer of smooth muscle cells), and adventitia with a small amount of connective tissue (Figures 1A-ND0w and 1B-D0w). The vein grafts taken from ND mice at 4 and 8 weeks showed significantly thickened vessel walls (e.g. up to 10 or 20 layers of cells and increased matrix protein accumulation) (Figures 1C-ND4w and 1E-ND8w). A marked increase in neointimal hyperplasia with various karyocytes was observed in the vein grafts from D mice (Figures 1D-D4w and 1F-D8w) relative to time-matched vein grafts from ND mice. Most of the cells in the vein grafts from both D and ND mice were smooth muscle cells (Figure S1). These results indicate that combined stimulation from rapidly increased blood pressure (hypertension) and hyperglycemia (diabetes) had a significant aggravated effect, promoting vein graft remodeling.

### Effects of combined increases in blood pressure and blood glucose on cell proliferation in mouse vein grafts

**Figure 2:**
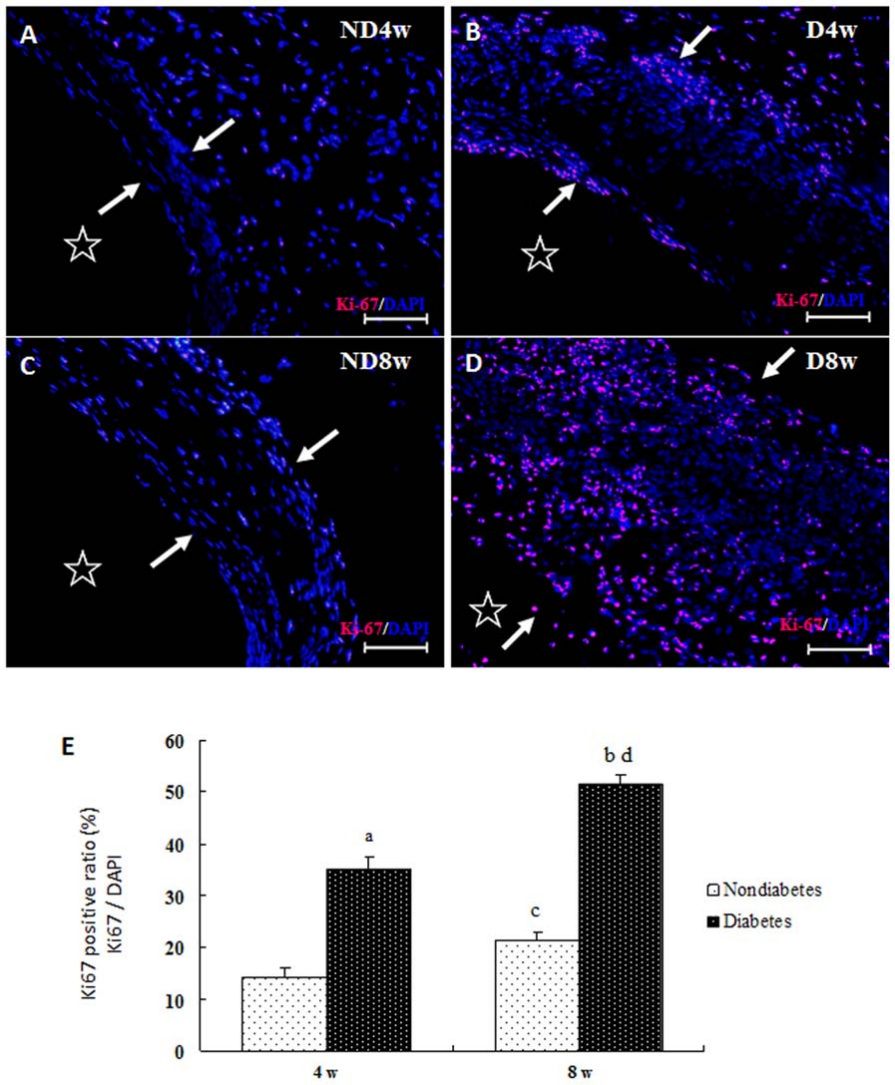
Representative photomicrograph of Ki-67 immunohistochemically stained sections of mouse vein grafts. Paraffin-embedded sections of the vein grafts of non-diabetic (Figures 2A-ND4w and 2C-ND8w) and diabetic (Figures 2B-D4w and 2D-D8w) mice killed 4 (Figures 2A-ND4w and 2B-D4w) and 8 (Figures 2C-ND8w and 2D-D8w) weeks after surgery were stained with primary Ki-67 antibody and TRITC-conjugated (red) secondary antibody and counterstained with 4′, 6-diamidino-2-phenylindole (DAPI) (blue). Arrowheads and asterisks indicate the wall thickness and lumens, respectively, of the vein grafts (Figures 2A–2D). The red symbols indicate the Ki-67 antigens, while the blue symbols indicate the nuclei of the vascular cells. Figure 2E shows a statistical graph of the proliferative ratio of the vessel wall cells in the vein grafts (mean ± SEM) obtained from seven animals per group at each point in time. a, b, c, and d, P<0.05 versus ND4w, ND8w, ND4w, and ND8w, respectively.

To determine whether the increased neointimal formation mentioned above is directly associated with increased proliferation of the vessel cells in intricate microenvironments, the vein grafts were stained with antibodies against Ki-67, which is preferentially expressed in the active proliferating cells but absent from resting cells (G0 phase). Results demonstrated that Ki-67-positive cells in the vein grafts of D mice (Figure 2B, 2D) were more numerous than in time-matched vein grafts from ND mice (Figure 2A, 2C). Notably, most of the Ki-67-positive cells were found in the adventitia of the vein grafts of ND mice at 4 and 8 weeks (Figures 2A-ND4w, and 2C-ND8w). More Ki-67 positive cells were found in the vein grafts from D mice than in grafts from ND mice, which were predominantly exhibited in the intima and adventitia of the vein grafts at 4 weeks (Figure 2B-D4w) and in all layers of the vessel wall at 8 weeks (Figure 2D-D8w). This indicates that increased blood pressure and hyperglycemia may aggravate vascular remodeling in different pathogeneses via increased vascular cell proliferation. Furthermore, the proliferative effect on cells closely linked to increased ERK phosphorylation in vein grafts (Figure S3), consistent with results in vitro described below.

### Effects of diabetic vein grafts on deposition of advanced glycation end products (AGEs) and expression of AGE receptors (RAGE)

**Figure 3:**
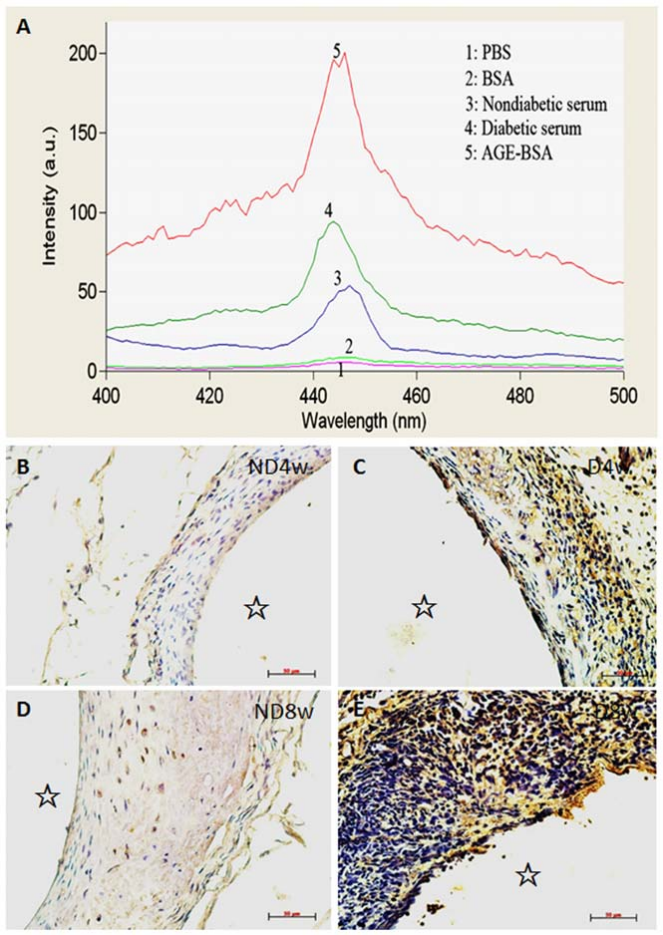
Identification of AGEs in serum (spectroscopic analysis) and vein grafts. Blood from the left atria of nondiabetic (Figure 3A-3) and diabetic (Figure 3A-4) mice was collected and the sera were separated for analysis of the characteristic fluorescence intensity pike of AGEs via spectroscopy. Dialyzed AGE-BSA came from bovine serum albumin (BSA) via incubation with high concentrations of glucose (0.5 M) for 8 weeks was used as a positive control (Figure 3A-5). PBS (Figure 3A-1) and BSA (Figure 3A-2) were used as negative controls. The characteristic fluorescence intensity pikes of AGEs in the diabetic serum were more than 2-fold relative to those observed in nondiabetic serum. Paraffin-embedded sections of the vein grafts from non-diabetic (Figures 3B-ND4w and 3D-ND8w) and diabetic (Figures 3C-D4w and 3E-D8w) mice killed 4 (Figures 3B-ND4w and 3C-D4w) and 8 (Figures 3D-ND8w and 3E-D8w) weeks after surgery were stained with primary AGE antibody and HRP-conjugated (brown) secondary antibody and counterstained with hematoxylin (blue). Significant AGE deposits (brown) were observed in the vein grafts of diabetic mice (Figures 3C-D4w and 3E-D8w). There were few brown deposits in the vein grafts of nondiabetic mice (Figures 3B-ND4w and 3D-ND8w). Bar = 50 µm.

Diabetes-induced vascular remodeling is closely associated with increased deposition of AGEs in tissues resulting from increased blood glucose. However, it is not clear whether increased AGE deposition occurs in vein grafts. As shown in Figure 3A, spectroscopic analysis indicated that characteristic fluorescence intensity of AGEs in serum from D mice increased more than 2 times relative to that in serum from ND mice. Immunohistochemical analysis also showed that a great number of AGEs were deposited on the vessel wall of the vein grafts of D mice (Figures 3C-D4w, and 3E-D8w), while very weak AGE signals were detected in ND mice (Figures 3B-ND4w and 3D-ND8w). Consistent with this, RAGE expression in the vein grafts of D mice was significantly increased relative to ND mice (Figure S2). These results imply that increased AGE/RAGE signals are closely associated with increased vascular cell proliferation in the vein grafts of D mice.

### Effects of combined stimulation via mechanical stretching and AGEs on activation of ERKs in VSMCs

**Figure 4:**
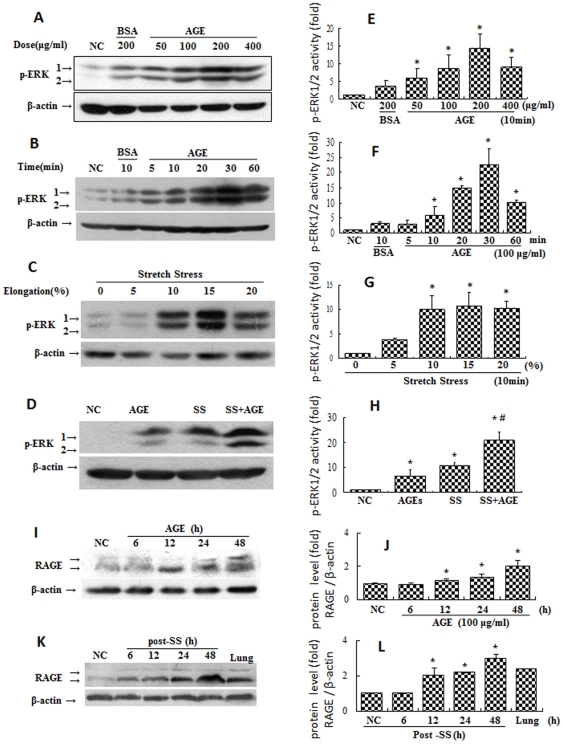
Effects of AGEs and mechanical stretching on phosphorylation of ERK1/2 in VSMCs. 80% confluent VSMCs were serum-starved for 48 hours and treated with AGEs and/or mechanical stretching as indicated for 10 minutes. They were then harvested for Western blot analysis. BSA was used as a negative control. Panels A and B show that AGEs induced ERK activation in VSMCs in a dose- and time-dependent manner. Panel C shows that mechanical stretching induced ERK activation in VSMCs in an elongation-dependent manner. Panel D shows that combined stimulation synergistically activated ERKs in VSMCs. Beta-actin served as an internal control. Graphs E, F, G, and H show the statistical results of phosphorylated-ERK (pERK) levels (ERK activity) corresponding to Panels A, B, C, and D from three independent experiments. Both (Panel I) incubation with AGEs or (Panel K) stimulation by mechanical stretching were found to elevate the expression of RAGE in a time-dependent manner. *P<0.05 *versus* negative control (NC), #P<0.05 *versus* AGE or stretch stress (SS).

Because VSMC proliferation plays a key role in neointimal formation in vein grafts, we investigated the molecular mechanism underlying the role of AGE/RAGE in VSMC proliferation. We evaluated the effects of mechanical stretching and AGEs on the activation of ERKs in VSMCs *in vitro* to imitate the vascular remodeling that occurs in diabetic patients with hypertension *in vivo*. Data showed that either AGEs or mechanical stretching alone could induce some phosphorylation of ERK1/2 in a time-, dose-, and elongation-dependent manner (Figures 4A, 4B, 4C). Furthermore, the level of ERK activation in the group treated with both mechanical stretching and AGEs was higher than sum of either single treatment (Figure 4D). These results suggest that the combined stimulation with mechanical stretching and AGEs may synergistically promote ERK activation.

### Effects of RAGE on activation of ERK1/2 induced by mechanical stretching and AGEs

**Figure 5:**
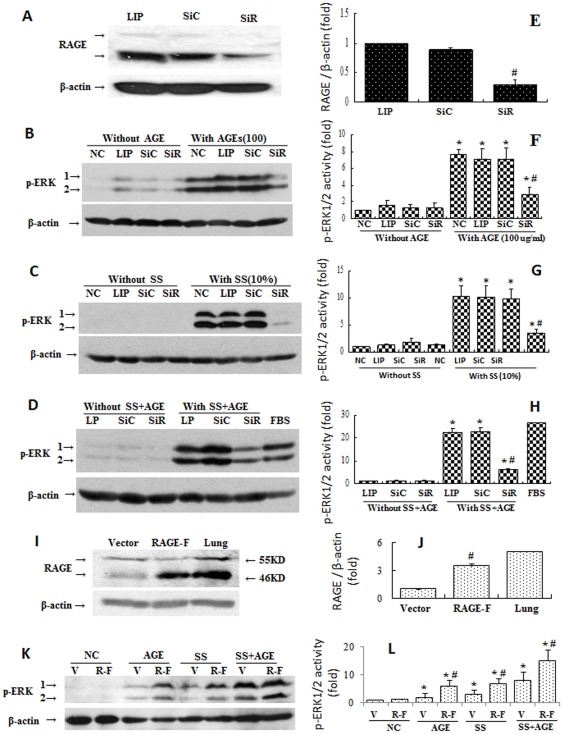
Effects of siRNA-RAGE and RAGE overexpression on the activation of ERK1/2 in VSMCs induced by stretch stress and AGE incubation. Panel A: Cultured VSMCs transfected by siRNA-RAGE (SiR) for 24 hours and then serum-starved for additional 48 hours were harvested. A siRNA-control (SiC) was used as a negative control. Graph E shows statistical results of RAGE expression from three independent experiments. *P<0.05 *versus* SiC. The siRNA-pretreated VSMCs were treated with AGE (Panel B) or cyclic stretch stress (SS) (Panel C) or both (Panel D) for 10 minutes. They were then harvested for detection of ERK phosphorylation levels. Graphs F, G, and H show the statistical results of phosphorylated-ERK (pERK) levels from three independent experiments. (I) Cells stably overexpressing RAGE were subjected to the same treatment and corresponding pERK was detected (Panel K). Graph L shows the statistical results of pERK levels of Panel K from three independent experiments.. *P<0.05 versus individual negative control (NC) without stimulation by AGE and/or SS; #P<0.05 versus SiC or vector within a given group. LIP represents Lipofectamine 2000. V represents cells transfected with empty vectors. R-F represents cells stably overexpressing full-length RAGE.

It remains to be seen whether RAGE is involved in the activation of ERKs induced by mechanical stretching with or without AGEs. As expected, RAGE expression was elevated by the presence of AGEs and by mechanical stretching (Figures 4I, 4J). Neither pharmacological RAGE inhibitors nor RAGE-deficient mice are commercially available, so we used siRNA-RAGE and RAGE over-expression assays to evaluate the contribution of RAGE to ERK1/2 activation. Figure 5A shows that VSMCs transfected with siRNA-RAGE showed specifically suppressed RAGE expression relative to siRNA controls. Significantly reduced levels of ERK phosphorylation were induced by mechanical stretching with and without AGEs (Figures 5B–5F). Over-expression of RAGE significantly amplified the intracellular signals initiated by mechanical stretching with and without AGEs relative to normal VSMCs (Figures 5I–5L). These data suggest that the process by which combined stimulation by mechanical stretching and AGEs synergistically promotes activation of ERKs is mediated via the RAGE signal pathway.

### Effects of RAGE on increased Ki-67 expression induced by mechanical stretching and AGEs

**Figure 6:**
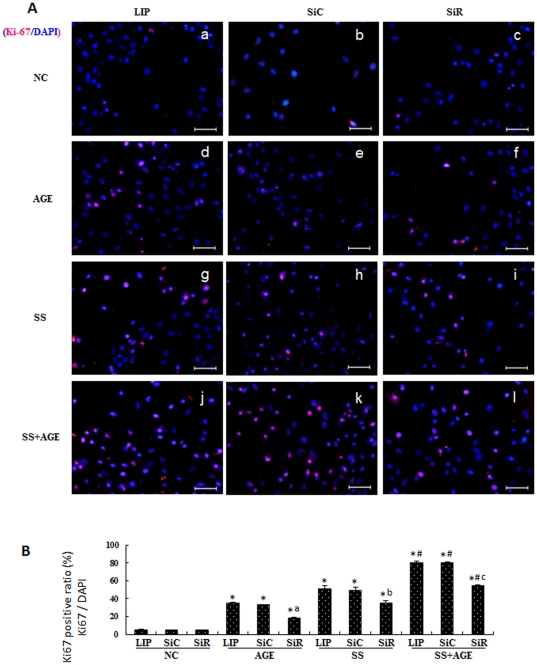
Effect of RAGE on VSMC proliferation (Ki-67 expression) induced by treatment of cyclic strain stress and/or AGEs. Cultured VSMCs were transfected by siRNA-RAGE (SiR) (Figures 6A-c, f, i, and l), siRNA control (SiC) (Figures 6A-b, e, h, and k) or Lipofectamine 2000 (LIP) (Figures 6A-a, d, g, and j) for 24 hours. They were then serum-starved for an additional 48 hours and treated with AGEs (Figures 6A-d, e, and f), cyclic stretch stress (SS) (Figures 6A-g, h, and i), or both (Figures 6A-j, k, and l) for 1 hour and cultured for another 24 hours. Figures 6A-a, b, and c show the negative control (NC). The cells were stained with primary Ki-67 antibody and TRITC-conjugated (red) secondary antibody and counterstained with DAPI (blue). The red symbols indicate Ki-67 antigens, while the blue symbols indicate the nuclei of the VSMCs. Bar = 50 µm. Figure 6B shows a statistical graph of ratio of Ki-67-positive cells from three independent experiments. *P<0.05 *versus* individual NC without stimulation of AGE and/or SS; #P<0.05 *versus* individual treatment in AGE or SS groups; a, b, and c P<0.05 *versus* SiC within a given group.

In the present study VSMCs transfected with siRNA-control (SiC) or siRNA-RAGE (SiR) were treated by mechanical stretching with or without AGEs, and then Ki-67 expression was detected by immunocytochemical staining. Results demonstrated that either AGEs (Figures 6A-d, -e, and -f) or mechanical stretching (Figures 6A-g, -h, and -i) alone could induce some increases in Ki-67 expression relative to negative controls (NC) (Figures 6A-a, -b, and -c). However, combined stimulation with both caused significant further increases in Ki-67 expression (Figures 6A-j, -k, and -l). The increased Ki-67 expression induced by mechanical stretching with and without AGEs was significantly inhibited by SiR (Figures 6A-f, -i, and -l) relative to NC (Figures 6A-d, -g, and -j) or SiC (Figures 6A-e, -h, and -k), which had no visible effects on Ki-67 expression in VSMCs. Figure 6B summarizes the statistical data related to the Ki-67 positive cell ratio. These results suggest that RAGE may mediate significantly increased VSMC proliferation induced by mechanical stretching with and without AGEs.

## Discussion

In diabetes patients and models, hyperglycemia may rapidly alter the already warped vascular cell proliferation profile induced by increased arterial blood pressure. This leads to accelerated neointimal formation in mouse vein grafts. This altered proliferation profile is closely associated with increased AGE deposition and RAGE over-expression in diabetic vein grafts. Our data show that combined stimulation by mechanical stretching and AGEs has significant synergistic effects on ERK activation and Ki-67 expression in VSMCs. While searching for an underlying mechanism we found that RAGE mediates not only the individual signals initiated by mechanical stretching and AGEs alone but also the combined signals induced by both. In this way, RAGE contributes significantly to the accelerated proliferation of VSMCs. These findings could significantly contribute to understanding of the potential effects of hypertension-mechanical stretching and diabetes-AGEs on vascular pathophysiology, suggesting a new role for RAGE in the pathogenesis of atherosclerosis.

Arteries exposed to sustained hypertension undergo marked intimal and medial thickening. The veins that are used as bypass grafts undergo similar histological changes [18], [25]. This is why vein grafts have been widely used as an indirect model of arteries exposed to sustained hypertension, and a direct model of venous bypass atherosclerosis in the diabetic setting [5]. Although a significant increase in neointimal formation was noted in the diabetic mouse vein grafts, no clear active cell proliferation profile could be established for either diabetic or non-diabetic vein grafts [5]. In this study, we found that rapidly increased blood pressure may cause fewer cells to express Ki-67 in the adventitia of the vein grafts in ND mice (15.1%). In diabetes, the active proliferating profiles of the vascular cells change markedly. Significantly increased numbers of active proliferating cells were found not only in the adventitia but also in the intima of the grafted veins at early stage (35.3%) (Figures 2A-D4w) and in all layers at a later stage (53.5%) (Figures 2A-D8w). This eventually led to rapidly increased neointimal formation (Figures 1A-D8w) relative to that of time-matched ND mice. These results seem to tell us that the vein grafts exposed to arterial blood pressure but not hyperglycemia may become arterialized vessels, while grafted veins exposed to arterial blood pressure with hyperglycemia would rapidly develop atherosclerosis.

The cause of the changed proliferation profiles of the vessel cells in the vein grafts in D mice remains unclear. Our results suggest that abundantly distributed AGEs in the serum and vessel walls interact with increased RAGE and are closely associated with the altered proliferation profiles. This is because numerous active proliferating cells appeared at various sites that showed obviously deposits of AGEs and elevated RAGE expression (Figures 3A-D4w, -D8w and 3B, and Figure S2). This indicates that rapidly increased blood pressure initiates cell proliferation in the adventitia of the grafted veins. Increased AGE deposition or RAGE expression may amplify these hypertension-induced signals and eventually change the proliferation profiles of the vessel cells. Hyperglycemia, AGE, and RAGE may play a pivotal role in promoting the development and progression of atherosclerosis via altered proliferation profiles of vascular cells in diabetic individuals. These results suggest that hyperglycemia management and RAGE inhibition are necessary before and after venous bypass graft surgery in clinical settings.

The migration, proliferation and differentiation of VSMCs are important to atherosclerosis. VSMCs are one of the most predominant cell types in the vein grafts (Figure S1) [5], [37], [38]. The means by which the VSMCs sense and transduce the signals initiated by mechanical stretching remains unknown. Previous *in vitro* studies have indicated that some receptors on cardiovascular cells, such as those on VSMCs, endothelial cells, and cardiomyocytes, can be directly activated by mechanical stretching [22], [26], [27]. These studies suggested that mechanical stretching may activate all the receptors on the cell membranes in a non-specific manner [22]. RAGE is a multiligand member of the immunoglobulin superfamily. It is expressed at low levels in vascular cells at homeostasis and is highly upregulated during vascular pathology (Figure S2) [39], [40]. RAGE activation in neointimal formation in arterial injury has been reported [41], [42], [43]. However, no prior reports are available concerning AGE deposition or RAGE expression in mouse vein grafts. In the present study, we proposed that the presence of AGEs may further amplify mechanical stretch-activated RAGE signals in vascular cells, accelerating pathophysiological consequences. Our results strongly support this hypothesis. Levels of AGE deposition, RAGE expression and ERK phosphorylation in D mice were found to be notably elevated (Fig. 3, Figure S2, and Figure S3) compared with that in ND mice, while either AGEs or mechanical stretching could increase RAGE expression in VSMCs (Figures 4I and 4J). Mechanical stretching and AGEs alone induced ERK activation and proliferation of quiescent VSMCs, but co-treatment with both triggered the highest levels (Figures 5–6). Stable over-expression of RAGE in VSMCs significantly amplified the above-mentioned effects (Figures 5I, 5J). In contrast, the suppression of RAGE expression *via* siRNA-RAGE transfection caused significantly decreased ERK activation and proliferation of quiescent VSMCs (Figures 5A through 5H and Figure 6). These results suggest that RAGE may mediate intracellular signals induced by mechanical stretching with and without AGEs, indicating a novel role for RAGE in vascular disease. Further study into RAGE and its downstream molecules may provide new targets for drug development.

Although the RAGE signal pathway plays a critical role in mediating signals induced by mechanical stretching and AGEs, other signal pathways also seem to affect VSMC proliferation. For instance, the suppression of RAGE expression in VSMCs with siRNA-RAGE transfection caused significant inhibition of VSMC proliferation induced by AGEs, mechanical stretching, or both, to a statistically significant degree in all three groups. However, siRNA-RAGE inhibited the proliferation rate of VSMCs in the AGE-alone group more than in groups treated by mechanical stretching or mechanical stretching with AGEs (Figure 6). One explanation is the simultaneous, nonspecific activation of multiple signal pathways in VSMCs initiated by mechanical stretching with or without AGEs [22], [23], [27], [28], [40]. Another possibility is a decrease in the efficiency of siRNA-RAGE due to too long a treatment time after mechanical stretching with and without AGEs. If either a RAGE inhibitor or RAGE-deficient mouse model were commercially available, inhibition of venous graft atherosclerosis related to RAGE signal transduction could be observed *in vivo*.

One limitation of this study is that the data collected from mouse materials cannot be precisely extrapolated to human clinical applications. However, these results should still be very useful for further clinical investigations. Another limitation of this study is that we were unable to characterize the cellular composition of the active proliferating cells, though we evaluated the proliferation index of the diabetic vein grafts relative to non-diabetic vein grafts. Based on HE-staining results, we found more cell types, including VSMCs, in the diabetic vein grafts. Using double-label immunocytochemistry, Hilker et al. found that the actively proliferating vessel cells in human bypass grafts mainly included VSMCs (α-actin), endothelial cells (CD 31), macrophages (CD 68), and T-lymphocytes (CD 45) as well as some unidentified cells [38]. We previously reported that the progenitor cells in the adventitia contribute to atherosclerosis of vein grafts in ApoE-deficient mice [37]. Shi et al. reported that adventitial myofibroblasts contribute to neointimal formation in injured porcine coronary arteries [44]. Which kinds of cell types contribute to the pool of actively proliferating cells in the vein grafts exposed to hypertension and hyperglycemia has yet to be fully determined. Finally, the ERK/Ki-67 signal pathway is a very stable and sensitive parameter of cell growth. It has been widely used in AGE-related studies. We introduced it into our study in order to emphasize the fact that RAGE mediates signals of mechanical stretching with and without AGEs. RAGE activation may initiate other important signal pathways related to vascular remodeling, such as the inflammation, migration, and apoptosis pathways, which need to be further investigated.

**Figure 7:**
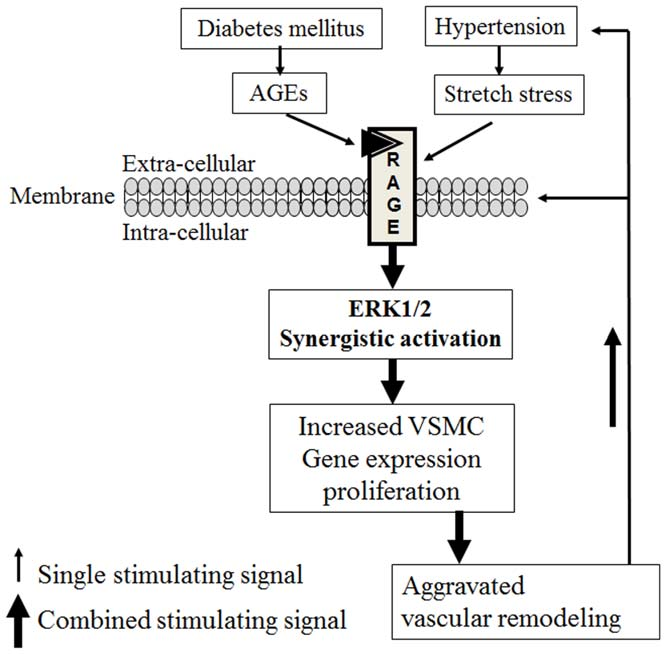
Role of the RAGE signal pathway in the synergistic effects of cyclic stretch stress and AGEs on ERK activation and proliferation in VSMCs. Increased blood pressure can trigger rapid increases in mechanical stretching on the walls of vein grafts. Stretch stress causes deformation of the vascular cells (VSMCs) and non-specifically activates RAGE and its downstream signal molecules, such as ERK, leading to over-proliferation (Ki-67 expression) of the vascular cells. Hyperglycemia can produce numerous AGEs. These modified proteins are deposited on the vascular wall, where they directly and specifically interact with RAGE and activate intracellular signaling molecules, altering vascular structure and function. Blocking RAGE and its downstream molecules may inhibit the synergistically accelerated vascular remodeling induced by hypertension-stretch stress with and without AGEs. Further investigations may provide new targets for drug development and new strategies for the treatment and prevention of vascular diseases, such as atherosclerosis, in diabetic patients.

In the present study we demonstrated that significantly accelerated neointimal formation in the vein grafts of STZ-induced diabetic mice mainly resulted from increased numbers of actively proliferating cells. This increase was initiated by both hypertension-mechanical stretching and diabetes-induced AGE deposition. This revealed the novel bridge role of RAGE in mediating intracellular signals induced not only by hypertension-mechanical stretching but also by AGEs. This led to synergistically increased ERK activation and proliferation of VSMCs and eventually accelerated venous bypass graft atherosclerosis (Figure 7). In this way, blocking RAGE may simultaneously decrease the deleterious effects of hypertension and diabetes. These findings and further studies (e.g. inflammation, migration, differentiation and apoptosis) may significantly advance current understanding of the potential effects of simultaneous hypertension-mechanical stretching and diabetes-induced AGE deposition on vascular pathophysiology. They may provide new drug targets and new therapeutic strategies against vein graft failure and primary vascular complications in hypertensive diabetes. This study closes the gap between basic research and clinical therapeutics (translational medicine).

## Supporting Information

Data S1 **An expanded methods section.**
(DOC)

Figure S1 **Predominant VSMCs in the vein grafts.** Paraffin-embedded sections of the vein grafts from (A, C) non-diabetic and(B, D) diabetic mice killed (A, B) 4 and (C, D) 8 weeks after surgery were stained with primary smooth muscle α-actin antibody and TRITC-conjugated (red) secondary antibody and counterstained with 4′, 6-diamidino-2-phenylindole (DAPI) (blue). Predominant VSMCs (red) were observed in the vein grafts. Asterisks indicate the lumens of the vein grafts. Bar = 50 µm.
(TIF)

Figure S2 **Increased expression of RAGE in the vein grafts from D mice.** Paraffin-embedded sections of the vein grafts from (A, C) non-diabetic and(B, D) diabetic mice killed (A, B) 4 and (C, D) 8 weeks after surgery were stained with primary RAGE antibody and TRITC-conjugated (red) secondary antibody and counterstained with 4′, 6-diamidino-2-phenylindole (DAPI) (blue). Significantly increased RAGE expressions (red) were observed in the vein grafts from diabetic mice. Asterisks indicate the lumens of the vein grafts. Bar = 50 µm.
(TIF)

Figure S3 **Increased phosphorylation of ERKs in the vein grafts from D mice.** Paraffin-embedded sections of the vein grafts from (A) non-diabetic and(B) diabetic mice killed 4 weeks after surgery were stained with primary phosphorylated-ERK antibody and TRITC-conjugated (red) secondary antibody and counterstained with 4′, 6-diamidino-2-phenylindole (DAPI) (blue). Significantly increased phosphorylation of ERKs (red) were observed in the vein grafts from diabetic mice. Asterisks indicate the lumens of the vein grafts. Bar = 50 µm.
(TIF)
