# Supplementary material for: RAGE Mediates Accelerated Diabetic Vein Graft Atherosclerosis Induced by Combined Mechanical Stress and AGEs via Synergistic ERK Activation
Source: PLoS One. 2012 Apr 9;7(4):e35016. doi: 10.1371/journal.pone.0035016 (PMC3322163; doi:10.1371/journal.pone.0035016)
Supplement: Data S1 — An expanded methods section. (DOC) [file pone.0035016.s001.doc]

**Supplementary Data 1 (Data S1)**

***Reagents***

Streptozotocin (STZ) (S0130), bovine serum albumin (BSA), monoclonal anti-actin, and α smooth muscle actin (SM-α-actin) (A5228) antibodies were purchased from Sigma. Polyclonal Ki-67 (sc-7846) and RAGE antibodies were purchased from Santa Cruz. Monoclonal phospho-p44/42 MAPK (ERK1/2) (#9106) and β-actin (#4970) antibodies were purchased from Cell Signaling Technology. Rabbit (ab6721) and mouse (ab6728) IgG secondary antibodies (HL) were purchased from Abcam. Polyclonal AGEs antibody was purchased from Abbiotec (San Diego, U.S.). Vectors with or without full-length RAGE were purchased from Origene (MC208117).

***Experimental animals***

All animal procedures were consistent with the National Institutes of Health Guide for the Care and Use of Laboratory Animals and approved by the Animal Care and Use Committee of Sun Yat-sen University. Procedures were similar to protocols described previously . In brief, three-month-old male C57BL/6J mice were purchased from the animal facility center of Sun Yat-sen University (Guangzhou, China) and maintained on a light/dark (12/12 h) cycle at 24°C and received food and water ad libitum before experimentation.

***Vein grafting of diabetic mice in vivo***

The mice were used as donors and recipients for vein grafts (N=50, respectively) and divided into a non-diabetic vein graft group and a diabetic vein graft group. The induction of experimental diabetes was similar to that described by Zauli . The recipients received seven consecutive daily intraperitoneal injections of 50 mg/kg streptozotocin (STZ) (Sigma) (diabetic mice, D mice) or citrate buffer (nondiabetic mice, ND mice) (N=25, respectively). Blood glucose concentrations were measured a week later and hyperglycemia (blood glucose level > 16 mmol/L) was confirmed in the diabetic group (N=24). The mice were subjected to vein graft surgery in a manner similar to that described previously . In brief, the right common carotid artery of the recipient was mobilized from the thoracic inlet to the bifurcation, divided at its midpoint, inverted over the polyethylene cuff and fixed with 8-0 silk sutures. The supradiaphragmatic vena cava from an isogenic littermate donor mouse was harvested and sleeved over the 2 cuffs and ligated as an interposition graft. Vigorous pulsations confirmed successful engraftment.

For histological analysis, perfusion was performed as described previously . After the mice were placed under anesthesia using pentobarbital sodium (50 mg/kg body weight, i.p.), blood was collected from each mouse’s left atrium for AGE measurement by spectroscopy (Varian, California, U.S.). Samples were perfusion-fixed with 0.9% NaCl and 4% paraformaldehyde. The vein grafts were harvested at 0, 4, and 8 weeks after the operation and paraffin-embedded (N=7, respectively) . Sample sections 7 mm thick were stained with hematoxylin and eosin (HE) and examined microscopically (Carl Zeiss, Oberkochen, Germany). The thickness of the grafted vessel wall, including the distance between the lumen intima and adventitia, was determined by measuring four regions of each cross-section.

***Immunohistochemical staining***

Procedures were in accordance with the protocols provided by Abcam (www.abcam.com/technical). Briefly, serial paraffin-embedded sections were stained with α smooth muscle actin antibody (1:200, Sigma-Aldrich, St. Louis, U.S.), AGE antibody (1:200, Abbiotec, San Diego, U.S.), or RAGE antibody (1:100, Santa Cruz, California, USA) or Ki-67 antibody (1:100, Santa Cruz) or phospho-ERK1/2 antibody (Cell Signal Tech., Inc., U.S.). The sections incubated with horseradish peroxidase (HRP)-conjugated secondary antibody were developed with chromogen (3,3′-diaminobenzidine, DAB) (brown) and counterstained with hematoxylin (blue). They were then inspected and photographed using visible light microscopy (Carl Zeiss, Oberkochen, Germany). The sections with TRITC-conjugated secondary antibody were counterstained with 4′, 6-diamidino-2-phenylindole (DAPI) (blue). They were inspected and photographed using fluorescence microscopy (Olympus BX51W1, Tokyo, Japan). Nuclei and Ki-67-positive cells were counted and analyzed. Active proliferating cells were identified by Ki-67-positive staining, and the proliferation index was calculated as the percentage of active proliferating cells versus the total cell count.

***Cell culture***

VSMCs were isolated by enzymatic digestion of aorta of C57BL/6J mice using a modification of the procedure as described previously . The isolated cells grown in silicone elastomer-bottomed, gelatin-coated 6-well culture plates were cultured in Dulbecco’s modified Eagle’s medium (DMEM) (Life Technologies, California, U.S.) supplemented with 10% fetal calf serum, penicillin and streptomycin at 37°C in a humidified atmosphere of 5% CO_2_.

***Preparation of AGEs***

AGEs were prepared in a manner similar to that described by Kim . In brief, 1 mM fatty acid-free BSA was dissolved in PBS with 0.5 M glucose and incubated under sterile condition for 8 weeks at 37°C. Reaction mixtures were dialyzed against phosphate-buffered saline (PBS) to remove free glucose and then passed through a specific column (Pierce, Illinois, U.S.) to remove any endotoxins. Non-glycated control BSA was subjected to the same conditions except that glucose was omitted. AGEs were identified by fluorescence spectrophotometry .

***Cyclic strain stress***

Treatment of VSMCs subjected to cyclic stretch stress was previously described . Briefly, VSMCs were plated on silicone elastomer-bottomed plates and gelatin-coated 6-well plates (Sigma-Aldrich, St. Louis, MO, U.S.; Flexcell, Meckeesport, PA, U.S.). Cells achieving 70% confluence were serum-starved for 48 hours and subjected to cyclic stretch stress using a computer-controlled modified cyclic stress unit. The unit was modified as described by Banes . The unit consisted of a controlled vacuum unit and a base plate to hold the culture plates (FX3000AFC-CTL, Flexcell). A vacuum (15 to 20 kilopascals) was repeatedly applied to the elastomer-bottomed plates via the baseplate, which was placed in a humidified incubator with 5% CO at 37°C. Cyclic deformation (60 cycles/min) achieving 5% to 20% elongation in elastomer-bottomed plates was performed with and without AGEs. The apparatus generates a homogeneous stretch stress on the membrane.

***Cell treatment***

For experiments of ERK phosphorylation, VSMCs grown in silicone elastomer-bottomed plates and gelatin-coated 6-well culture plates to 80% confluence were serum-starved for 48 hours and subjected to treatment with AGEs (50–400 μg/ml), cyclic stretch stress (elongation 5–20%), or cyclic stretch stress (10% elongation) in the absence or presence of AGEs (100 μg/ml) for 10 minutes. They were then harvested for protein extraction and Western blot analysis. For observations of the effects of RAGE small interfering RNA (siRNA-RAGE) on ERK phosphorylation and Ki-67 expression in VSMCs, siRNA-RAGE-treated or -untreated VSMCs grown in silicone elastomer-bottomed plates and gelatin-coated 6-well culture plates to 80% confluence were serum-starved for 48 hours and subjected to treatment with cyclic stretch stress (10% elongation) in the absence or presence of AGEs (100 μg/ml) for 10 minutes. They were then harvested for protein extraction and Western blot analysis or cultured for an additional 24 hours for immunocytochemical staining. We used procedures provided by Origene to establish stable VSMC lines expressing RAGE.

***Western blot analysis***

The procedures using in present study were similar to that we previously described with slight modification [[29](#_ENREF_4)]. Treated or untreated VSMCs were washed twice with ice-cold PBS and harvested by scraping on ice in buffer A, containing 50 mM β-glycerophosphate, 20 mM Hepes (pH 7.4), 2 mM EGTA, 1 mM Na_3_VO_4_, 1 mM DTT, 1% Triton X-100, 10% glycerol, and complete protease inhibitor cocktails (Roche, Switzerland). The suspension was incubated on ice for 30 minutes with votexing every 5 minutes. It was then centrifuged at 12,000 rpm for 20 minutes at 4°C. The supernatants were separated, and protein concentration was assessed using a Bio-Rad protein assay (Bio-Rad Laboratories, Hercules, CA, U.S.). After heating for 10 minutes at 100°C, equal amounts of denatured protein were resolved by 12% SDS-PAGE and electrophoretically transferred onto nitrocellulose membranes. The blots were probed with antibodies specific to the phosphorylated forms of ERK1/2 and RAGE. Membranes were stripped and reprobed with antibody against β-actin as an internal control. The bands were visualized using an enhanced chemiluminescence (ECL) detection system (GE Healthcare) and their intensity was quantitated by densitometry.

***RNA interference***

Procedures were similar to those described by Villacorta et al . The following RAGE small interfering RNA (siRNA-RAGE) target duplex sequences [NM_007425] were used: sense: 5'-GAGACACCCUGAGACGGGACUCUUU-3'; antisense: 5'- AAAGAGUCCCGUCUCAGGGUGUCUC-3'. These siRNA duplexes were synthesized by Invitrogen (U.S.). A non-targeting siRNA duplex sequence (Invitrogen Stealth^TM^ RNAi) was used as a negative control. VSMCs were seeded at a density of 5×10^4^ cells per well into silicone elastomer-bottomed, gelatin-coated 6-well plates and grown in DMEM containing 10% fetal calf serum for 24 hours. They were then transfected 100 pmol of the siRNA duplexes for each well using Lipofectamine 2000 and Opti-MEM according to the manufacturer’s recommendations. After 24 hours of transfection, the cells were serum-starved for an additional 48 hours and then subjected to treatment with cyclic strain stress (10% elongation) in the absence or presence of AGEs (100 μg/ml) for 10 minutes. They were then harvested for detection of ERK phosphorylation. For observations of the effect of siRNA-RAGE on Ki-67 expression, the VSMCs treated above were cultured for an additional 24 hours and subjected to immunocytochemical staining. Negative control experiments were performed using equivalent amounts of non-targeting siRNA.

***Statistical analysis***

All analyses were performed with SPSS 16.0 (SPSS Inc, Chicago, U.S.). Continuous variables are given as mean ± SEM and were analyzed by ANOVA. Categorical variables are given as actual numbers and percentages and were analyzed by chi-square testing. There were no missing values. P values were adjusted for multiple comparisons of data with either the Scheffé or modified Bonferroni method. Findings were considered statistically significant at the 0.05 level.
