# Supplementary figures and images for: RAGE Mediates Accelerated Diabetic Vein Graft Atherosclerosis Induced by Combined Mechanical Stress and AGEs via Synergistic ERK Activation
Source: PLoS One. 2012 Apr 9;7(4):e35016. doi: 10.1371/journal.pone.0035016 (PMC3322163; doi:10.1371/journal.pone.0035016)

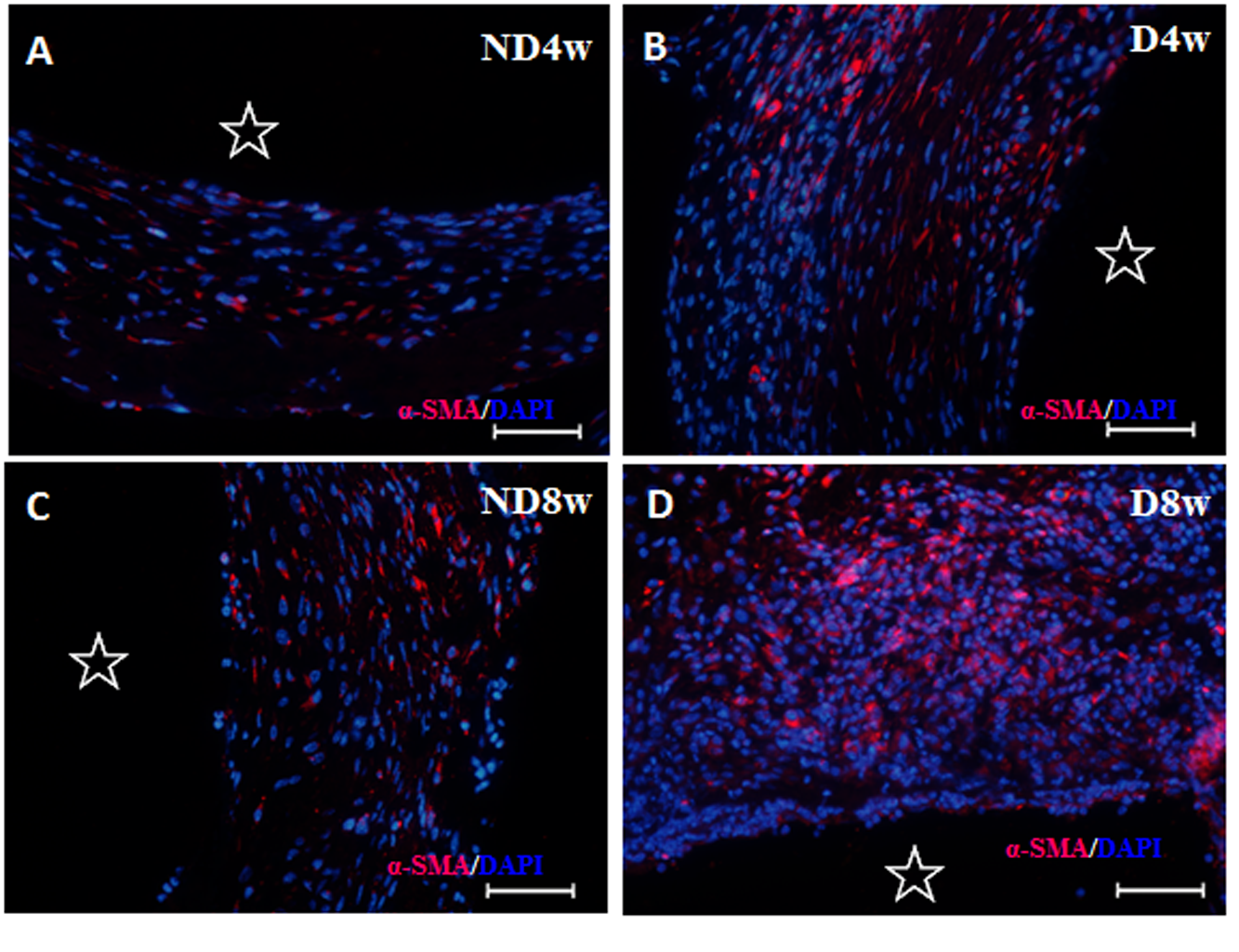

Supplement: Figure S1 — Predominant VSMCs in the vein grafts. Paraffin-embedded sections of the vein grafts from (A, C) non-diabetic and(B, D) diabetic mice killed (A, B) 4 and (C, D) 8 weeks after surgery were stained with primary smooth muscle α-actin antibody and TRITC-conjugated (red) secondary antibody and counterstained with 4′, 6-diamidino-2-phenylindole (DAPI) (blue). Predominant VSMCs (red) were observed in the vein grafts. Asterisks indicate the lumens of the vein grafts. Bar = 50 µm. (TIF) [file pone.0035016.s002.tif]

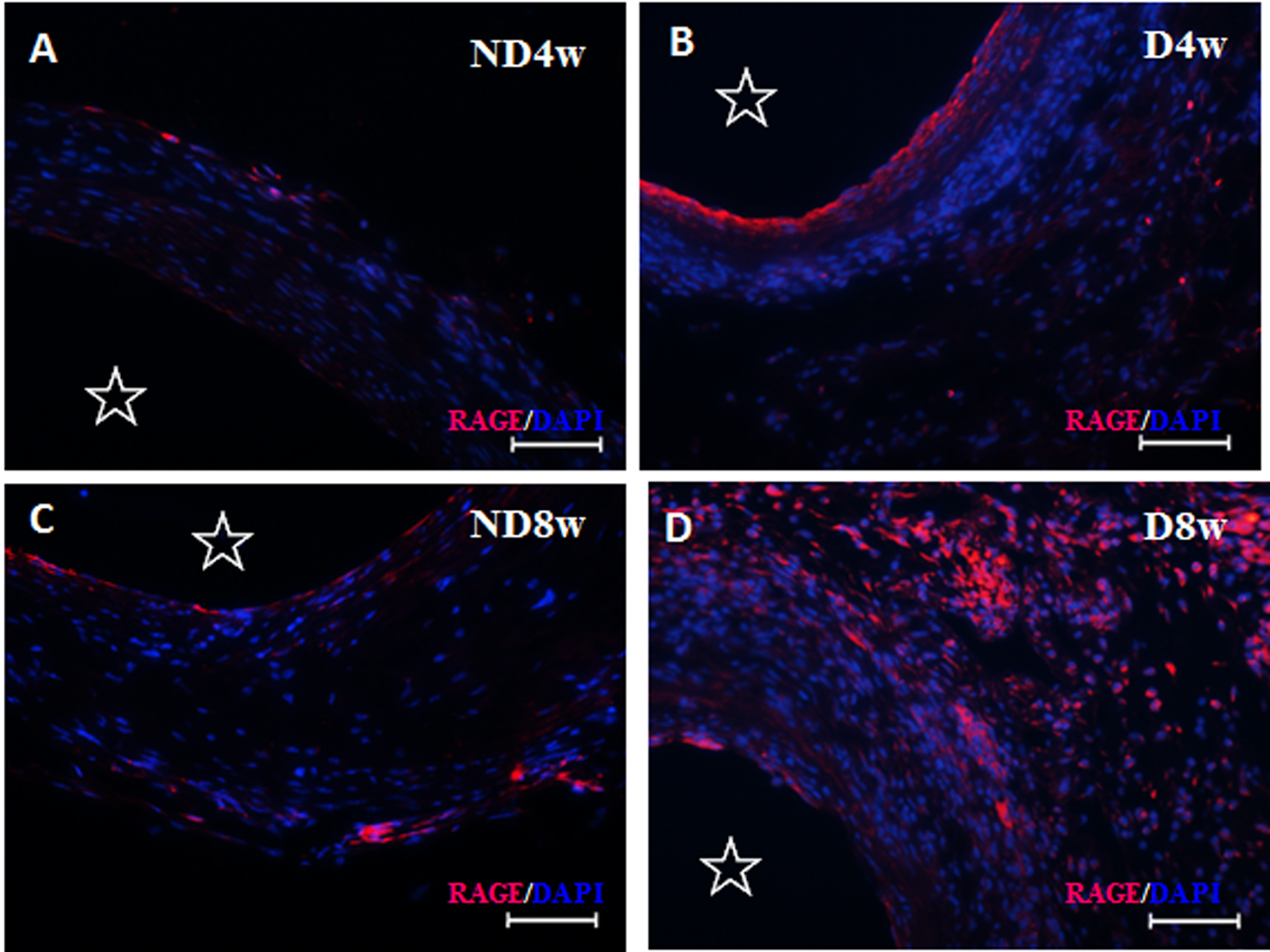

Supplement: Figure S2 — Increased expression of RAGE in the vein grafts from D mice. Paraffin-embedded sections of the vein grafts from (A, C) non-diabetic and(B, D) diabetic mice killed (A, B) 4 and (C, D) 8 weeks after surgery were stained with primary RAGE antibody and TRITC-conjugated (red) secondary antibody and counterstained with 4′, 6-diamidino-2-phenylindole (DAPI) (blue). Significantly increased RAGE expressions (red) were observed in the vein grafts from diabetic mice. Asterisks indicate the lumens of the vein grafts. Bar = 50 µm. (TIF) [file pone.0035016.s003.tif]

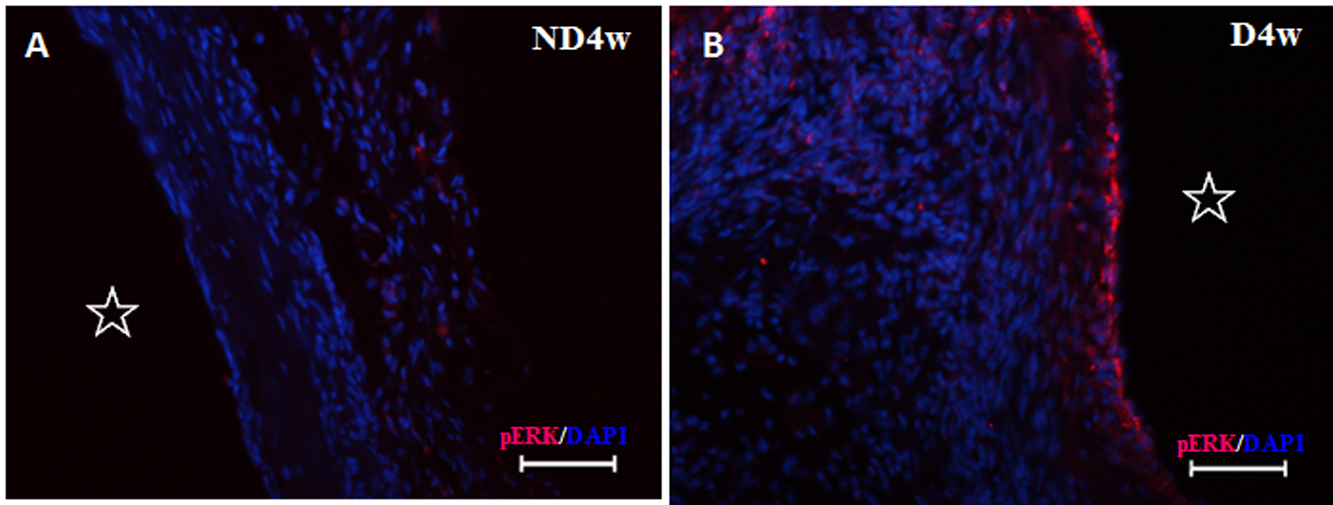

Supplement: Figure S3 — Increased phosphorylation of ERKs in the vein grafts from D mice. Paraffin-embedded sections of the vein grafts from (A) non-diabetic and(B) diabetic mice killed 4 weeks after surgery were stained with primary phosphorylated-ERK antibody and TRITC-conjugated (red) secondary antibody and counterstained with 4′, 6-diamidino-2-phenylindole (DAPI) (blue). Significantly increased phosphorylation of ERKs (red) were observed in the vein grafts from diabetic mice. Asterisks indicate the lumens of the vein grafts. Bar = 50 µm. (TIF) [file pone.0035016.s004.tif]
